# Perceived threat and fear responses to e-cigarette warning label messages: Results from 16 focus groups with U.S. youth and adults

**DOI:** 10.1371/journal.pone.0286806

**Published:** 2023-06-23

**Authors:** Rosemary J. Avery, Motasem Kalaji, Jeff Niederdeppe, Alan Mathios, Michael Dorf, Sahara Byrne, Amelia Greiner Safi

**Affiliations:** 1 Jeb E. Brooks School of Public Policy, Cornell University, Ithaca, NY, United States of America; 2 Department of Communication Studies, California State University, Northridge, Los Angeles, CA, United States of America; 3 Department of Communication, Cornell University, Ithaca, NY, United States of America; 4 Department of Economics, Cornell University, Ithaca, NY, United States of America; 5 Law School, Cornell University, Ithaca, NY, United States of America; 6 Department of Public and Ecosystem Health, Cornell University, Ithaca, NY, United States of America; University of Wisconsin-Madison, UNITED STATES

## Abstract

**Aims:**

A warning on e-cigarette packaging is one way the U.S. government can inform the public of known harms of e-cigarette use. Currently, the only required warning on these products is: “WARNING: This product contains nicotine. Nicotine is an addictive chemical.” This exploratory study aims to inform potential future investigations and FDA decisions regarding alternative warnings that may generate fear in addition to being intentionally informational.

**Method:**

Data were obtained from responses by 16 online focus groups with adult (N = 47, age range = 18–64) and youth (N = 32, age range 14–16) participants with various smoking and vaping experiences. We showed each focus group a set of hypothetical e-cigarette warning labels to determine how they respond to currently existing public statements that communicate information on the toxicity of ingredients in e-cigarettes, potential health risks, addiction to nicotine, and the uncertainty of the science regarding health effects of using these products. The focus group interviews were audio recorded and transcribed. Transcripts were subjected to a multiphase coding process to identify common response themes. Codes derived from the Extended Parallel Processing Model were then applied to understand impact of potentially fear-inducing language by warning category and age group.

**Results:**

For adults, all warnings—except those about addiction—gave rise to spontaneous danger control (intended) responses, such as quit intentions. Warnings highlighting cognitive and uncertain effects may be particularly promising for adult consumers of tobacco products because both gengerated danger control and response efficacy without evidence of fear control. However, responses also suggest that warnings risk discouraging some adults who use combustible cigarettes from transitioning to e-cigarettes for harm reduction. For youth, while evidence of response efficacy and danger control emerged among youth exposed to messages in all warning categories but one–addiction—unproductive reactions indicative of fear control were also prevalent among youth respondent across most warning types. On average, youth were more skeptical than adults about the harms of using e-cigarettes.

**Policy implications:**

Implications of study findings for the development of future effective e-cigarette warning messages are explored.

## Introduction

Mandated warnings on e-cigarette packaging and advertising are one way the U.S. government can accomplish three parallel–and occasionally conflicting–goals: to inform the public of known harms and potential risks of tobacco and nicotine-containing products; to discourage product use initiation; and, to reduce harm to people who currently smoke combustible cigarettes by encouraging them to switch completely to e-cigarettes [1–3].

The U.S. Food and Drug Administration (FDA) currently requires manufacturers, distributors, and advertisers to include the following warning on e-cigarette products and advertisements: “WARNING: This product contains nicotine. Nicotine is an addictive chemical” [4]. FDA consideration of potential new, additional, or alternative warnings for these products is complicated by the fact that e-cigarettes are relatively new products on the market, and the evidence of their health effects is still being generated [5]. The development of potential new FDA warnings for e-cigarette products faces the challenge of communicating emerging knowledge about product risks along with uncertainty about the existence and likelihood of both short- and long-term health effects [6]. Product warnings also would need to balance the potentially competing goals of discouraging youth and people of all ages who do not smoke from vaping while encouraging people who currently smoke combustible cigarettes to switch to e-cigarettes as harm reduction or a pathway to cessation [7, 8].

In developing new warnings for e-cigarettes, the FDA will need to weigh several lines of evidence: 1) what is known about the absolute risk of using e-cigarettes; 2) the relative risk in comparison to cigarettes–as e-cigarettes do pose harm reduction potential for those who switch completely or substantially reduce their combustible cigarette smoking in favor of e-cigarettes [9, 10]; and 3) the associated impact of warnings on different audiences based on age and their smoking/vaping habits.

Given the relative novelty of vaping products, determining all of the health effects of using e-cigarettes, especially the longer-term effects, will take time. Some of the risks of e-cigarettes are already well-established. These include risks of addiction, possible developmental consequences of exposure to nicotine, and effects of exposure to carcinogenic ingredients or byproducts that have been found in some e-cigarettes and e-cigarette aerosols [11]. Furthermore, the presence, dosing, and concentration of nicotine and other chemicals in e-cigarettes vary substantially among different products/brands, and the exact product components are often unclear, further complicating the assessment of risk impact [12]. The nicotine content of e-cigarettes is especially concerning for U.S. youth, who had been adopting e-cigarettes at an alarming rate through 2020. From 2018 to 2019, current use of e-cigarettes, defined as having vaped in the past 30 days, increased from 20.8 percent to 27.5 percent percentamong U.S. high school students and from 4.9 percent to 10.5 percent among U.S. middle school students [13, 14]. However, vaping decreased substantially in 2021 to 11.3 percent of U.S. high school students and 2.8 percent of U.S. middle school students [15], perhaps as a temporary result of the pandemic that kept many students at home under the watchful eye of a parent and away from the social influence of others and opportunities to vape outside the home. Recent data from 2022 indicate only a slight increase from the 2021 levels, with 14.3 percent of U.S. high school students and 3.3 percent of middle school students reporting current vaping [16].

This study aims to inform potential future FDA decisions regarding how warnings might elicit fear responses in tadem with three additional goals: (1) communicating risk to those currently using e-cigarettes; (2) discouraging use among those who do not smoke or vape (using the current science connecting ingredients to negative health effects); and (3) encouraging people who smoke to switch substantially or completely to e-cigarettes (to realize harm reduction benefits from the e-cigarettes). We conducted a series of focus group interviews to understand how youth and adults with various smoking and vaping experiences respond to hypothetical warning statements that communicate information on the toxicity of ingredients in e-cigarettes, potential health and developmental risks, risk of addiction to nicotine, and the uncertainty of the science regarding health effects of using these products. Informed by the Extended Parallel Processing Model (EPPM) [17, 18], we pay particular attention to aspects of the warnings that might elicit: (1) fear emotions and actions to avoid harm (danger control); (2) responses to control fear that may be induced by the warning (fear control); and (3) beliefs about the validity of the claim in the warnings and whether the interpretation is accurate or not (response efficacy/inefficacy). This analysis complements a parallel paper that focuses on various forms of uncertainty conveyed by candidate warning label messages tested in the same study [19].

## Background

### Government and health association statements concerning the health effects of e-cigarettes and harm reduction

Much remains unknown about the long-term health consequences of e-cigarettes. Still, the connection between ingredients found in e-cigarettes/aerosols and the potential harmful health impacts of those ingredients is being widely reported by the Centers for Disease Control and Prevention (CDC), the American Heart Association, and the American Lung Association. Nicotine product use has been reported to have detrimental effects on the lungs [20], to have detrimental developmental effects on developing fetuses [21], and to have negative effects on the brain development of youth [22, 23]. Furthermore, e-cigarettes can contain or produce other harmful substances besides nicotine; e-cigarette aerosols may hold the potential for lung damage [22–25]. However, the long-term health effects of vaping are difficult to predict and would likely vary by age of initiation, and the extent of the exposure to nicotine, other ingredients, and byproducts [26]. Research on e-cigarettes is complicated by the fact that many different devices are being sold, with different concentrations of nicotine and with a range of possible ingredients [27].

### E-cigarettes and harm reduction

The negative effects of e-cigarettes on the body should be viewed alongside the potential benefits of these products as a harm reduction tool for people who smoke combustible cigarettes [28]. The adverse health consequences of combustible tobacco use have been documented extensively [29], and a major goal of tobacco control efforts is to reduce their health burden [30]. Accordingly, when complete cessation is not feasible, federal health agencies suggest strategies that minimize or reduce but do not eliminate harm from smoking, and e-cigarettes offer people who smoke a potential harm reduction alternative [12]. If people who smoke switch to specific approved e-cigarette products, they can significantly reduce their exposure to harmful and potentially harmful constituents (HPHC), in comparison to what they would encounter in combustible cigarettes [31].

### Effectiveness of e-cigarette warnings

Limited data exist on the effects of the FDA’s 2016 required e-cigarette warning–and alternative warning messages that have been employed in other contexts–on perceptions of both absolute and relative health risk of e-cigarettes in populations of interest to inform potential future regulatory activity. The results of existing research have been mixed, with earlier studies finding limited impacts on risk perceptions [32] and more recent research shedding light on warning content that might influence perceptions and behavior. Below, we first discuss warnings components that we expected to hold potential to elicit perceived threat and fear, based on theory and previous research in tobacco and other domains.

### Effectiveness of various warning content

The current FDA-required warning focuses on nicotine and addiction. However, the research on the efficacy of addiction theme warnings has been mixed. For instance, one study using an addiction-focused warning found no difference in how risky e-cigarettes were perceived (perceived harm, addictiveness) between ads with and without the addiction-focused warnings [33]. Contrary to these findings, other studies have shown that addiction-focused warnings can be effective in changing consumers’ risk beliefs [34, 35] and lowering vaping susceptibility [36].

Still other studies find information regarding addiction less persuasive than information regarding toxins or harmful chemicals. For instance, a study by Wackowski, et al. (2019) randomly assigned young adults (ages 18–29) to view e-cigarette ads with a variety of themes (e.g., nicotine addiction; nicotine’s impact on adolescent brain development; presence of harmful chemicals). In comparison to the current FDA nicotine warning, the adolescent-specific and chemical-specific warnings were deemed more effective in discouraging youth from using e-cigarettes [37]. Similarly, another study found that chemical and brain warning messages were rated higher than nicotine warning messages in terms of use deterrence, and that knowledge, thoughts, and beliefs about e-cigarette harms increased/strengthened after exposure to these warnings [38].

In a study that included messages related to uncertainty about harms of product use, Owusu, Massey and Popova (2020) found that ’uncertainty about ingredients’ failed to elicit higher risk perceptions when compared to the control condition (nicotine and addiction) [39]. In a survey with youth focused on the framing of consequences of use (a loss frame) or benefits of non-use (a gain frame), Kong et al. (2016) found that emphasizing consequences of use was more effective in increasing risk perceptions when the topic was related to health risks, addiction, or being labeled as a person who smokes, while emphasizing benefits of non-use was more effective when the topic was financial (e.g., money saved by not vaping) [40].

### Advancing the public health function of warnings

Several empirical questions remain regarding warnings’ impact on product use, including quitting. From a public health perspective, the goal is to find a way to design warnings effectively enough so that: 1) people who do not smoke–especially children and adolescents–do not start vaping or smoking; 2) those who have used only e-cigarettes understand this as an activity that involves risks; and 3) the warnings do not deter people who smoke combustible cigarettes from switching to e-cigarettes. Fear-based approaches may or may not be a pathway to these goals, as they possess the potential to backfire [41, 42] and may also result in judicial invalidation as an infringement of manufacturers’ free speech if government requires them to include messages that instill fear rather than neutrally informing consumers of risks [43, 44].

Given the dearth of evidence on adult and youth reactions to e-cigarette warnings, our study addresses how warnings with different health risk messages are perceived by audiences in terms of beliefs, intentions, and perceived susceptibility. This study examines transcripts from 16 focus group interviews undertaken with adults and youth. Our goal is to assess how specific warning content (harmful chemical ingredients, negative health consequences, impact on cognitive development, addiction, and uncertainty about safety) may shape respondents’ perceived danger, fear/emotional responses, attitudes, and behavioral intentions regarding e-cigarette use.

### Methods

The data for this analysis were collected July to August 2020 as part of a broader study examining advertising and warnings on e-cigarette products. The study involved 16 focus groups conducted via an online platform (Adobe Civicom) because of the state of the Covid-19 pandemic at the time. A commercial research firm [45], with expertise in qualitative methods, conducted the interviews based on a protocol provided by the study authors. They conducted 8 focus groups with youth (n = 32, grouped by self-reported sex to increase comfort in participation) and by whether they used e-cigarettes before or not) and 8 mixed-gender adult focus groups (n = 37, grouped by those who smoke, those who use both e-cigarettes and cigarettes (dual use), and those who had previously smoked combustible cigarettes but had switched to e-cigarettes). All study protocols were approved by the Cornell University Institutional Review Board. All youth provided verbal assent after their parents/guardians consented; all adult participants provided verbal consent. Each of the focus groups lasted approximately 120 minutes. Each focus group participant received compensation of $100 for their participation.

### Focus group composition

The adult sample consisted of two groups of adults who dually used combustible cigarettes and e-cigarettes, two groups of adults who formerly smoked combustible cigarettes and switched to e-cigarettes only, and four groups of adults who currently smoked combustible cigarettes (but not e-cigarettes). The youth sample consisted of two separate groups of females and males who had used/tried e-cigarettes before (but not combustibles), and two separate groups of males and females who had never tried/used e-cigarettes and combustible cigarettes.

The adult sample had a mean age of 44 (SD = 12.1, range = 18–67). 68 percent of the adult sample were female, 57 percent non-Hispanic White, 27 percent Black/African American, 13 percent Hispanic, and 3 percent Asian. In terms of education, 43 percent had a college degree or more, and 57 percent had some college or less education. Forty-three percent of adult sample participants had an annual household income of $75,000 or less. Mean age of the youth sample was 14.6 years (SD = 0.5m range 14–16), 53 percent were female, 72 percent were non-Hispanic white, 28 percent Black/African American, 19 percent Hispanic, and 6 percent Asian. Thirty-five percent of youth lived in households earning $75,000 or less. Full detail of study methods, participant recruitment, and study materials and protocols are reported in a parallel paper that focuses on responses to message cues that convey various forms of uncertainty [19].

### Participant recruitment

Participants were selected based on demographic and other criteria that the research team established to support an inclusive set of focus groups given variation in risk of initiation by income, race, and education [46–49]. We also grouped participants based on their experiences with use of cigarettes and e-cigarettes. Youth were recruited so that some focus groups had youth without e-cigarette experience and others had youth who were using e-cigarettes. Adults who use tobacco products were grouped based on their use of various products (people who smoke combustible cigarettes only, people who vape e-cigarettes, and people who dually use combustible cigarettes and e-cigarettes). We required that all groups be racially and/or ethnically diverse, that participants have a minimum household income of $25k/year, and that they have no affiliation with the tobacco or research industries. We also required that all adult participants have at least a high school diploma because the warnings were text-based and thus required basic literacy.

### Experimental warning statement development

We collected publicly available e-cigarette warning statements from public health sources such as the FDA, the Truth Campaign, the CDC, and the Surgeon General. The warning messages collected from these sources were organized into five groups, each identifying specific risk aspects of e-cigarette use: toxic substances in these products; potential health effects of using the product; impact on youth cognitive development; addictive effects of nicotine use; and/or future uncertainty about the effects of e-cigarettes on the body (Table 1).

**Table 1:** Experimental warning statements.

Type of Warning	Warnings
Toxic Ingredients (6 warnings)	1. Vapor from some e-cigarettes can deliver toxic metal particles like lead, chromium, and nickel into your lungs. 2. This product can deliver toxic metal particles into your lungs. 3. Vapor from some e-cigarettes can expose you to toxic chemicals like formaldehyde that may cause lung damage. 4. This product may expose you to toxic chemicals that may cause lung damage. 5. Some e-cigarette vapors contain the chemicals formaldehyde, acrolein, and acetaldehyde—also found in cigarette smoke—which can cause irreversible lung damage 6. This product may contain some of the same toxic chemicals found in cigarette smoke which can cause irreversible lung damage
Health Effects (2 warnings)	7. Some e-cigarette vapors contain harmful chemicals that may cause cancer, central nervous system problems, and irreversible lung damage. 8. This product may pose health risks to young people.
Cognitive development (2 warnings)	9. Exposure to nicotine during the teenage and young adult years may harm the developing brain, which may have long-term effects on memory, attention, and mood. 10. Vaping e-cigarettes can harm the teenage and young adult brain.
Addiction (4 warnings)	11. Vaping e-cigarettes can expose you to the highly addictive chemical nicotine. Some vape pods contain as much nicotine as 20 cigarettes. 12. The nicotine in e-cigarettes can prime the teenage and young adult brain for addiction to other drugs such as cocaine. 13. Vaping e-cigarettes during the teenage and young adult years is strongly linked to the use of other tobacco products [such as regular cigarettes and hookah]. 14. Using nicotine in the teenage and young adult years can increase risk for future addiction.
Unknown risks (3 warnings)	15. The full extent of the safety and harms of this product have not been confirmed by FDA-approved research 16. Current evidence is insufficient to recommend e-cigarette use for tobacco cessation in adults, including pregnant women. 17. Research is uncertain on whether e-cigarettes are effective for quitting smoking

**Note:** Four additional experimental warnings, included in the larger study were not included in this analysis. These four warnings focused on a comparison between combustible cigarettes and e-cigarettes and switching behavior.

We focus our analysis in this paper on a subset of 17 warnings, excluding four that compare similarities and differences in the content of combustible cigarettes and e-cigarettes, for which results will be reported elsewhere in a forthcoming paper [19].

### Focus group procedures

In consultation with C+R Research (https://www.crresearch.com/methods), we created a discussion guide to explore adult and youth perceptions of, and reactions to, the warning statements we developed. This study focuses specifically on participant responses to language in these warnings regarding the ingredients in e-cigarettes and the health impacts on the body. The full discussion guide is available in S1 Appendix. Study discussion guide.

Each group of participants saw eight warning statements to evaluate. The text of the warning statements was presented on the shared screen and read aloud to participants. The warnings were not presented on packaging. Focus groups were randomly assigned to view warning statements from each of the categories, and each focus group saw at least one warning statement from each category during their interview. Each focus group, therefore, responded to eight randomly assigned warnings. At no time were respondents specifically asked about fear, meaning that any responses regarding fear or related contstructs were spontaneously generated by participants.

Table 1 includes the 17 warning statements analyzed here, and the category into which each was classified. Every focus group saw at least one statement from each category, and each statement was seen at least once by every demographic segment. All the focus group interviews were audio-recorded, professionally transcribed, and checked for accuracy. The research team used the verbatim transcripts to classify and analyze participant reactions to the warnings.

### Qualitative analytic approach

The analysis of the focus group transcripts followed the best practices of generic qualitative analysis and pragmatic qualitative analysis, in that we were addressing a current real-world problem of how to best understand the impacts of e-cigarette warnings [50–52] across different priority audiences and by different warning types. Our analysis involved a two-step approach. First, we pursued a thematic analysis of all responses to warning labels from the parent study. With this inductive approach, we allowed themes to emerge from the data, as we did not have preconceived ideas of what we would code for or particular theoretical models to apply or test. As is typical in qualitative research, the research team began informal analysis while observing the focus groups in July and August of 2020. We met as a team after the first round of focus groups and as often as possible over the two weeks the focus groups were conducted. In our initial thematic analysis, we applied many tools of thematic analysis and grounded theory [53]. We repeatedly read through the transcripts and noted common themes. Differing reactions to warnings that might induce fear and uncertainty were two of the prominent themes from the parent study. We write about uncertainty in a separate manuscript [19]. For this manuscript, we explored the many reactions to warnings that might indicate fear.

For the second step, we turned to existing literature. We focused in particular on finding established theoretical frameworks we could apply that would both help us make sense of reactions to potentially fear-inducing messages and give us a way to identify policy-relevant trends about the interaction among warning content and possible implications for future behavior related to e-cigarettes. Toward this end, we employed the Extended Parallel Processing model [17, 18] to inform our analysis of the results by helping to categorize participant responses to the warnings. In this second step, we moved from an inductive to a more deductive approach as we assigned all responses to one of four possible codes: 1) Danger Control Responses; 2) Fear Control Responses; and 3) Response Efficacy; or 4) Response Inefficacy. Each response received one of the four designations, which are described below and in Table 2.

**Table 2:** Definitions of concepts in the Extended Parallel Processing Model (EPPM).

EPPM Concept	Concept Definition
Danger Control	Stimuli that change attitudes and behavior to avoid the danger. A response was coded as “Danger Control” if the response to the warning indicated intentions to control the danger (e.g., intentions to avoid using the product, intentions to quit using the product).
Fear Control	Stimuli containing high threat messages that generate attitudes and behavior to control the danger. A response was coded as “Fear Control” if the warning generated one of three responses: (a) denial of the validity of the threat; (b) psychological reactance (expressed counterarguments to the one presented in the warning); and (c) defensive avoidance (disregard for the message impact on self or others).
Response Efficacy	A response efficacy code was applied to focus group participant statements affirming the validity of the claim in the warning and acknowledging that heeding the warning will reduce the risk.
Response Inefficacy	A response inefficacy code was applied to focus group participant statements indicating that the message was inaccurately interpreted or otherwise misconstrued.

### Participant code 1: Danger control responses

According to the EPPM [17, 18] if an individual perceives the severity of the communicated message to be high, they are likely to act to control the danger (danger control). A response was coded as “Danger Control” if the response to the warning indicated intentions to control the danger (e.g., intentions to avoid using the product, intentions to quit using the product).

### Participant code 2: Fear control responses

The EPPM [17, 18] further suggests that warnings perceived as containing high threat messages do not necessarily encourage people to control the danger. Instead of motivating one to reduce the threat (danger control) these messages can instead induce fear and cause a participant to control that fear. A response was coded as “Fear Control” if the response: 1) *denied* the validity of the message; 2) indicated *defensive avoidance (*reactions in which message recipients disengage from the message, including stating that the message will have no impact on self or others); or 3) demonstrated *psychological reactance*, which involves counterarguing, perceiving manipulative intent, or dismissing or diminishing the issue or the credibility of the message source.

### Participant codes 3 and 4: Response efficacy/inefficacy

The final coding categories derived from the EPPM are response efficacy and response inefficacy. A “Response Efficacy” code was applied to focus group participant statements affirming the validity of the claim in the warning and acknowledging that heeding the warning will reduce the risk. Conversely, a “Response Inefficacy” code was applied to focus group participant statements indicating that the message was inaccurately interpreted or otherwise misconstrued. Neither is behavior-focused; these solely attend to beliefs about the message.

### Categorizing participant responses to warning content

After coding the reactions according to the schema described above, we organized responses by the specific warning type that elicited them, with the aim of making the findings as policy-relevant as possible. As noted above, the warnings included here fall under five content domains: toxic ingredients; health impacts; impact on cognitive development of youth; addiction; and unknown risks. The final set of excerpts included representative quotes from the various groupings of adults who smoke (people who dually use combustible cigarettes and e-cigarettes, people who smoke combustible cigarette only, people who switched from combustible cigarettes to e- cigarettes) as well as male and female youth focus group responses (those who used and never used e-cigarettes).

Within each of the five warning content categories, the researchers reviewed the applied codes to derive general observations about focus group participants’ responses to viewing the message content. After this preliminary assessment, the team met to review the assigned codes and associated summaries until agreement was reached about the results. In the results section below, we compare responses across warning content categories and, when possible, between adult and youth or by smoking/vaping status.

## Results

We categorized our coding of participant responses (Table 2) by warning type (i.e., toxins, health effects, impacts on cognitive development, addiction, and unknown risks). We compared adult and youth reactions and compared participant responses by smoking experience (and, for the youth focus group, by gender). A summary of study findings is presented in S2 Appendix. Summary of results.

### Toxins warnings

These warnings (Warnings 1–6 in Table 1) delivered messages regarding toxic chemicals in e-cigarettes and suggested irreversible lung damage, cancer, and central nervous system problems as being potential effects of using these products. Among adults, the chemical names and health consequences communicated in these warnings were seen as alarming, generating fear emotions, and seen as a deterrent to using these products. The wording in the warnings referring to potential “irreversible lung damage” and “cancer” were identified as particularly fear inducing. Examples of these **danger control** responses included:

- “*It’s already convincing*, *this puts the fear and the scaredness in me*.*”* (Adult who smokes combustible cigarettes only)
- “*The word ’irreversible’ and the word ’cancer’*, *those two words right there making me really stop and question whether or not I want to do this*.” (Adult who dually uses e-cigarettes and combustible cigarettes)

After seeing warnings identifying specific toxic ingredients in these products, many adult participants concluded that e-cigarettes “are no better for you” than combustible cigarettes even though such a comparison was not included in the warning. Specific mention of the toxic ingredients in e-cigarettes elicited an undesirable conclusion from adults. Several adults concluded that e-cigarettes are as dangerous as combustible cigarettes, when e-cigarettes are known to be a harm reduction alternative to combustible cigarettes. Such **response inefficacy** statements included:

- “(*What this product) will deliver is worse than cigarettes can deliver*.” (Adult who dually uses e-cigarettes and combustible cigarettes)
- “*I liked that it shows that it’s also found in cigarette smoke*. *So*, *they’re showing you once again*, *this really isn’t a safe*, *alternative to cigarette smoke*. *It’s the same*.” (Adult who formerly smoked combustible cigarettes who switched to e-cigarettes only)

The fact that some adult participants viewing these warnings concluded that e-cigarettes are just as dangerous as combustible cigarettes is likely to work against the public health goal of encouraging people who smoke to switch to a less harmful product.

Warnings that claimed irreversible lung damage from exposure to toxic ingredients generated counterarguments among adults who smoke combustible cigarettes. The adults who smoke combustible cigarettes presumably comprised the most vulnerable group of focus group participants in terms of irreversible lung damaged most closely associated with cigarette smoking, and it is not surprising that their reactions to this type of warning were indicative of fear, eliciting a rejection of the message itself. Such a reaction is a marker of the **psychological reactance aspect of fear control**. An example of such a reaction is:

- “*I don’t believe that it’s irreversible*. *I think it’s irreversible to a certain point*, *but I think if you quit*, *younger*, *you will heal*.*”* (Adult who smokes combustible cigarettes only)

Messages identifying the toxic nature of these products and identifying unfamiliar chemical names of the ingredients in e-cigarettes generated strong **fear emotions and danger control responses among youth,** including those who have tried and those who have not tried e-cigarettes in the past. Many of these toxic ingredient names would be unfamiliar to the general public, and the unfamiliarity was particularly scary to the youth. These types of warnings might be particularly effective in discouraging youth from using these products. Examples of their responses about what stood out about the warning included:

- “*Lead*, *toxic metal particles*, *because I think that’s just super scary*, *and nobody wants that in their body*. *So that would make me want to avoid it as much as I can*.*”* (Youth Female–Has not tried cigarettes or e-cigarettes)
- “*I liked how it told specific things that can enter your lungs if you do smoke e-cigarettes and I feel that would scare people into not wanting to do it*” (Youth Female–Used/tried e-cigarettes only)

The use of these unfamiliar chemical names also lent credibility to the warning amongst the youth. **Response efficacy** was present in their remarks such as:

- *“Chemical names… It makes it seem a lot more dangerous*.*”* (Youth Male–Has not tried cigarettes or e-cigarettes)

Some male youth participants (both those who have tried, and those who have not tried e-cigarettes) viewing these warnings, however, expressed counterarguments to the message, the **psychological reactance form of fear control**. Male youth participants were, in general, more likely to develop counterarguments to warnings across all categories of warnings we examined. Some examples of these statements were:

- *“Well*, *it says "can" expose you*, *so that doesn’t really phase me*. *It’s not ensuring that you’re going to get this lung damage “(*Youth Male–Used/tried e-cigarettes only)
- *“Because there’s only some things that are irreversible*. *The damage to lungs*, *that can be redone after two or more years of nonsmoking or not being near those who smoke*.*”* (Youth Male–Has not tried cigarettes or e-cigarettes)

A few male youths who used e-cigarette viewing these warnings believe they would not be read or even heeded by young people and would therefore not be effective in persuading youth not to use these products. It is unclear from their statements below whether they are referring to themselves, or beliefs about other youth in their demographic group. Such statements exhibit the **defensive avoidance aspect of fear control** and included examples such as:

- “*But it’s again like kids that are going to smoke*, *they’re not going to look at something like if it’s a whole sentence*, *they’re not going to think to read it*. *They’re not even going to look at it*. *They’re just going to open the packaging and smoke it*.” (Youth Male–Used/tried e-cigarettes only)
- “*Yeah*, *my opinion I’ve talked to kids that smoke often before*, *and I’ve told them that you can’t like*… *It could hurt your lungs*, *but they just shrug it off*. *Like they don’t really care about it*.*”* (Youth Male–Used/tried e-cigarettes only)

### Health effects warnings

Warnings in this category (Warnings 7–8 in Table 1) mentioned potential health effects of using e-cigarettes, including the development of cancer, central nervous system damage, and irreversible lung damage. As previously noted with respect to adult participant reactions to toxic ingredient warnings, among some adults these warnings led to the incorrect conclusion that e-cigarettes are as harmful to health as combustible cigarettes, even though such a comparison was not included in the warning. As this is misinterpreting the warning, these responses are indicative of **response inefficacy** and could lead to undesirable behavior such as continued adherence to the use of combustible cigarettes. For example, adult participants who dually used e-cigarettes and combustible cigarettes noted:

- “*I just think that regular cigarettes cause cancer and lung damage too*, *so it’s six of one*, *half a dozen of the other*. *It’s up to you which one you want to do*.” (Adult who dually used e-cigarettes and combustible cigarettes)
- *“But that’s the same as cigarettes*. *The same thing about cigarettes*, *cigars*. *They’re giving you the option*, *that you have to be at your own discretion on that*.” (Adult who dually used e-cigarettes and combustible cigarettes)

As with some of the previous responses, these warnings of health effects from using e-cigarettes could lead to the *unintended consequence* of discouraging people who smoke combustible cigarettes from switching to e-cigarettes or impacting views of relative safety of these products among those who use e-cigarettes. These types of reactions among adults who dually use combustible cigarettes and e-cigarettes could undermine the public health goal of reduced harm from reducing combustible use in this population.

These reactions were not universal, however. Warnings with “cancer” and “irreversible lung damage” in their text elicited a desired reaction among some adult participants of intentions to quit, indicative of **danger control:**

- “*I felt like it communicated the seriousness associated with smoking really well to the point where I was like*, *Okay*, *if I looked at this*, *this might make me stop smoking*, *just this statement*.” (Adult who formerly smoked combustible cigarettes who switched to e-cigarettes only)

In addition, a few adult focus group participants (those who use e-cigarettes) mentioned that, despite the serious health consequences of using these products identified in the warnings, they did not believe these warnings would have an impact on their own product use or quitting behavior. This belief is an example the **defensive avoidance component of fear control** and suggests that these warnings might have only a minimal impact on current e-cigarette use behavior among adults. Given the known addictive nature of nicotine, this result is not surprising. Examples of these statements from the focus group participants were:

- *“It might have someone rethink their decision*. *But if you’re going for it*, *these warnings don’t really matter*.*”* (Adult who formerly smoked combustible cigarettes who switched to e-cigarettes only)
- *I mean if you’re going to do some*… *In the broad things that I’ve done*, *I don’t think out of all the information that I’ve known*, *did I say*, *you know what*? *that warning made me not do it*.*"* (Adult who formerly smoked combustible cigarettes who switched to e-cigarettes only)

It is encouraging to note that some youth viewing these warnings found them to be powerful and scary, suggesting they might hold potential to deter initiation of product use or cause them to stop using these products altogether. Examples of these **danger control reactions were:**

- *“I would be super scared*. *Which is good because it would stop me from using e-cigarettes”* (Youth Female–Used/tried e-cigarettes only)
- “*Well*, *that would stop me from doing it more because cancer*, *nervous*, *all those things*.*”* (Youth Female–Has not tried cigarettes or e-cigarettes)
- “*Because it makes you think*. *And maybe you shouldn’t do that because it could cause damage to your body*.*”* (Youth Male–Used/tried e-cigarettes only)

However, some male youth who have not tried e-cigarettes developed counterarguments to these warnings, suggesting that there is not enough evidence to support the validity of these claims, thereby diminishing the warnings’ potential to deter behavior. These are examples of the **psychological reactance component of fear control:**

- “*Wasn’t really that convincing to me*. *I think more scientific evidence was needed*.*”* (Youth Male–Has not tried cigarettes or e-cigarettes)
- *“I feel like there should be more evidence maybe*.*”* (Youth Male–Has not tried cigarettes ore-cigarettes)

For warnings that stated generic “health risk to young people,” some youth respondents noted that this is a message they had heard many times before and it was no longer impactful on their vaping decisions. Language in a warning statement like this might exemplify warnings that youth have heard from their parents many times before and that they have started tuning out has a defensive mechanism. These were examples of the **defensive avoidance version of fear control**:

- “It’s *saying pretty much something we all know*. *I’m pretty sure anyone that smokes*, *knows that it’s not good for them…*… *I’m pretty sure everyone knows that by now*.” (Youth Male–Has not tried cigarettes or e-cigarettes)
- *“Boring*…… “*Mainly*, *that you just constantly keep hearing this*. *You don’t tell us what’s going to happen*. *We know that it’s a health risk*, *but is that going to change anybody*?” (Youth Female–Used/tried e-cigarettes only)

Some male youth participants viewing warning statements claiming ‘health risk to young people’ incorrectly inferred that these products do not offer risks to other older demographic groups, indicating **response inefficacy.** It is not clear how such response inefficacy can be averted with a warning statement. Examples of male youth respondents’ statement in this regard were:

- “*The one thing that was weird to me*, *was how it said young people*, *and so it made me feel like maybe it does not affect older people*.” (Youth Male–Has not tried cigarettes or e-cigarettes)
- “*Just saying ‘young ‘people’ makes me think that older people won’t be affected by the product”* (Youth Male–Has not tried cigarettes or e-cigarettes)

### Cognitive development warnings

Warnings in this category (Warnings 9–10 in Table 1) contained language indicating that the use of e-cigarettes during the teenage years might harm the developing brain and have long-term effects on memory and mood. These warnings had a strong youth focus but were seen by participants in all age groups. Even though these warnings did not mention adults, adults who viewed these warnings still had strong emotional reactions to these warnings, focusing on wanting to protect youth from e-cigarette use. Perhaps due to their age and experience, adults often found these statements to be highly credible and believed such warnings would be a strong deterrent to people considering using these products. These types of responses signified **danger control and response efficacy,** at least among adult population:

- “I *think that may create a cause for a pause for somebody considering the nicotine products*.*”* (Adult who smokes combustible cigarettes only)
- “*I think it’s very convincing*, *if a teenager or young adult bought one of these things and read that in there*.” (Adult who smokes combustible cigarettes only)

Among youth participants, warnings regarding the impacts on brain development, long-term memory, and mood were accepted as true, showing widespread **response efficacy** to such warning messages and holding potential for impacting use behavior:

- “*It mentioned the brain and how it can have long lasting negative effects on you*, *so that was just really scary to think about*.*”* (Youth Female–Has not tried cigarettes or e-cigarettes)
- “*I find that it’s really true because you’ve seen teens that are smoking and they’re having trouble in school*,… *Honestly*, *with the teens I know that is true*.” (Youth Female–Used/tried e-cigarettes only)

Supporting the power of these types of warnings, some youth believed that this warning might act as a deterrent to young people initiating e-cigarette use **(*danger control*)**. An example of such a response was

- *“*… *If a teenager would be reading this*, *I don’t think he’d want to do it because*… *I mean*, *as it can say*, *if you’re a teenager*, *it has*… *It could harm you in multiple different ways*, *worse than adults*. *Because first*, *your brain is undeveloped*, *and it could have long-term memory impacts like it says on it*. *That’s*… *I don’t know*, *you won’t be able to remember as much stuff*.*”* (Youth Male–Used/tried e-cigarettes only)

However, several female youth participants noted fatigue around the messaging that linked e-cigarette use and memory, noting that they had heard these types of claims repeatedly from adults. They responded with statements indicating that such messaging no longer impacted them. Such reactions show the **defensive avoidance form of fear control**.

- *“And I just feel that I’m sort of tired*… *It’s been told to me so many times it almost holds no meaning anymore*. (Youth Female–Used/tried e-cigarettes only)
- *“But in the same breath*, *we hear that all the time*. *And let’s be honest*, *no 13*, *14*, *15-year-old cares about the long-lasting effects on their memory and attention and mood*. *We just don’t care*. *I don’t know why*. *Because we’re teenagers*. *So*, *we’re just going to read that and be like*, *"Okay*.*"* (Youth Female–Used/tried e-cigarettes only)

### Addiction warnings

Warnings in this category (Warnings 11–14 in Table 1) asserted that the nicotine in e-cigarettes is a highly addictive chemical that can prime the young brain for addiction to other drugs such as cocaine, lead to the use of other tobacco products such as hookah, and can increase the risk for future addiction overall. The claim that nicotine is a highly addictive product has been well-established, but the relationship between the use of nicotine and future addiction to other substances has not. However, adults in our focus groups expressed beliefs that e-cigarettes can lead to “future addiction,” a sign of **response efficacy** to the warning message:

- *“… ‘future addiction’ is a true statement*.*”* (Adult who smokes combustible cigarettes only)
- *“Because I feel this statement is completely true*, *because when you start with e-cigarettes*, *and I do feel that teenagers and young adults*, *it will lead them to go on to regular cigarettes*. *… I do believe that if you go from one drug you will go to another*, *cigarette is a drug*.” (Adult who smokes combustible cigarettes only)

However, some adults in our focus groups noted that linking smoking addiction to cocaine addiction raised concerns about the appropriateness of the comparison in an e-cigarette warning, **a psychological reactance form of fear control**. The same adults thought the comparison to be harsh and inappropriate overall. One adult noted:

- *“So*, *when you said*, *the comparison on it*, *cocaine kills people*. *People overdose on cocaine*. *Cocaine destroys families*. *It’s a horrible comparison*. *Can’t do that*.*”* (Adult who smokes combustible cigarettes only).

Youth participants, both male and female, generally saw such warnings as convincing, a sign of **response efficacy**, and concluded e-cigarettes could be worse than combustibles for their addictive potential and believed the suggested link to cocaine to be impactful. Some examples of the statements were:

- “*It’s this telling us about it could be taken to cocaine*. *Everyone knows that cocaine is really bad and addicting and cocaine could easily lead to other drugs*.” (Youth Male—Used/Tried e-cigarettes only)
- “I *think that it’s really good because of how it kind of tells you that nicotine is a gateway drug*, *how that’s why people are so scared of weed because they thought it was the gateway drug leading to harder drugs”* (Youth Male—Used/Tried e-cigarettes only)
- “*…*. *because it’s saying*… *It’s kind of all or nothing*, *in a way*. *Most likely*, *you’re not just going to have one thing*. *It’s probably going to lead to worse things*, *which is warning you of so many things*, *which is scary* (Youth Female–Never used combustible cigarettes or e-cigarettes)

On the other hand, in response to warnings claiming potentially addictive qualities of e-cigarettes and the suggestion that their use might lead to addiction to other more harmful products, several youth participants indicated that they had already heard these types of messages repeatedly and expressed the belief that the repetition of these messages in a warning might not be heeded by young people and would not deter them from using the product, indicating the **defensive avoidance version of fear control**. Some examples of these expressed beliefs were:

- “*Because the first thing seems like it’s addictive*, *yeah*. *But I’m pretty sure everyone knows that*, *but they wouldn’t care because they’re already addicted to it*” (Youth Male—Used/Tried e-cigarettes only)
- “*But I didn’t really like*… *the future addiction part; you’re so young*. *You’re not going to be thinking that far to your future*. *You’re just going to be like*, *"You know what*? *I’m a kid I’m using this now*. *The outcome isn’t too*… *It’s all in the back of my head*. *It’s not even climbing its way to the front at all*. *It’s just sitting there"*. (Youth Female- Used/Tried e-cigarettes only)

In response to warnings suggesting that e-cigarettes might lead to future addiction to other substances, a few female youths also displayed **defensive avoidance** in their skepticism about the addictive potential and stated that they did not believe the claim that e-cigarettes would be a gateway drug:

- “*I think it’s just not everything leads to bigger drugs or some things that are not as big as that stuff*. *Like e-cigarettes doesn’t mean someone’s just going to go do cocaine right after*. *Not really*.” (Youth Female- Used/Tried e-cigarettes only)
- “*Sure*, *it could be a gateway drug*, *or you start with cigarettes and sure they can help other addictions*. *But just because I’m doing this doesn’t mean I’m going to be doing other things*.*”* (Youth Female—Used/Tried e-cigarettes only)

### Unknown risks warnings

Warnings in this category (Warnings 15–17 in Table 1) emphasized the uncertainty of the science in this product category, noting that the full extent of the safety and harms of these products has not been confirmed by FDA-approved research and that current evidence is insufficient to recommend e-cigarettes as a smoking cessation product. Statements regarding the uncertainty of the science surrounding the benefits and harms of e-cigarettes raised concerns among adult focus group participants. Adult focus group participants found these warnings to be scary, and raised questions about their health effects, **a form of response efficacy:**

- "*It’s because it has not been confirmed by the FDA"*, *that kind of bothers me*. *And I know that it took years for cigarettes to be deemed harmful and everything*, *but it is kind of scary to not know what’s going to happen in 10 years with me or with anybody who is smoking the e-cigarettes”* (Adult who formerly smoked combustible cigarettes who switched to e-cigarettes only)
- “*Personally*, *not knowing what’s going to happen in the next 10 years*, *not knowing what the effects are going to be because I was a smoker and then I switched over to vaping*… *The combination of that*, *what’s it going to do*? *And so*, *it kind of opened my eyes*. *It’s scary not realizing what’s going to happen and it has not been confirmed*.*”* (Adult who formerly smoked combustible cigarettes who switched to e-cigarettes only)

Female youth participants differed from both adult participants and male youth participants in their response to these types of uncertainty warnings. Female youth expressed **danger control** statements in noting that the uncertainty about impacts to one’s body might be an effective deterrent to using these products:

- *“But honestly*, *if I knew that it wasn’t fully researched about it*, *why would I want to put it in my body because I don’t know what could happen*.*”* (Youth Female–Used/tried e-cigarettes only)
- *“Just feel it’ll prevent people from smoking the product because I mean*, *of course*, *if you don’t know how something is going to fully affect you concerning your health*, *I would assume that you wouldn’t risk it or test it because you’re not sure*.*”* (Youth Female–Used/tried e-cigarettes only)

These uncertainty warnings also elicited some undesirable conclusions, particularly among male youth participants. In contrast to adults and female youth participants, a few male participants incorrectly concluded that if effects have not been identified so far, the products were probably safe, another case of **response inefficacy.** An example of such a statement was

- *“I feel like it doesn’t help at all because I understand it’s supposed to make you scared because if the FDA*… *When a bunch of scientists don’t know*, *then how are you supposed to know what it’s going to do for you*? *But I think most people would just take that as*, *"Oh*, *they haven’t found any bad things about it*, *so there’s nothing bad about it*.*"* (Youth Male–Used/tried e-cigarettes only)

## Discussion

For adults, all warnings—except those about addiction—gave rise to spontaneous danger control (intended) responses, such as quit intentions. Warnings highlighting cognitive and uncertain effects may be particularly promising for adult consumers of tobacco products because both generated danger control and response efficacy without evidence of fear control. However, responses also suggest that warnings risk discouraging some adults who use combustible cigarettes from transitioning to e-cigarettes for harm reduction.

Fear-evoking messages in warnings have been explored in other studies using cigarette warning labels, particularly pictorial warning labels. Fear-evoking warning labels have been linked with desired message outcomes such as higher intention to quit smoking (***danger control***) [54–57] and higher perceived message effectiveness (**response efficacy**) and intentions to quit smoking (***danger control***) [55, 58, 59]. Overall, among adult focus group participants, warnings mentioning the toxic ingredients in e-cigarettes, and suggesting unknow risks of using these products appear to hold significant promise for effective communication of risks.

The finding that, among adults in our sample, all but one of our warning categories (addiction), gave rise to ***danger control*** responses, such as intentions to quit using the product is consistent with previous research. Similar responses to fear inducing warning messages were found in a study by Mead et al. (2016). More participants in this study were motivated to call the quit line following exposure to a high threat message [60]. In turn, these adaptive ***danger control responses*** also have been associated with positive attitudes towards fear-inducing warnings. For example, in a study by Owusu and colleagues (2019), participants who reported danger control responses (65 percent) following exposure to a high-threat warning reported higher perceived informativeness of warning label compared to participants who reported maladaptive responses (only 15 percent) [61].

**Fear control** responses such as ***defensive avoidance*** and ***psychological reactance*** emerged in response to most warnings (toxins, health effects, and addiction) though no evidence for fear control reactions was observed in response to warnings about impacts on youth cognitive development and unknown risk of using these products. Some adult participants who smoke combustible cigarettes developed counterarguments to claims of irreversible lung damage from smoking e-cigarettes (***psychological reactance*)**, and some adults who vape e-cigarettes indicated that warnings about the health effects of e-cigarettes on the body would not necessarily impact their use behavior (***defensive avoidance)*.** It is not surprising that adults in our study who smoke cigarettes and e-cigarettes counterargued claims of harm on lungs from e-cigarettes. Some research suggests that adults who smoke and/or vape have lower e-cigarette related health and addiction risk beliefs than those who do not smoke or vape [35, 62–64]. Other research suggests that adults who smoke might have lower risk perceptions in general. For instance, Owusu et al., (2019) found that non-smoking adults and those who intend to quit were more likely to report danger control responses than adults who smoke following exposure to a cigarette warning label [61]. Similar findings are reported in an experimental study with smoking and non-smoking young adults. Participants who smoke reported less fear-related reactions and were less discouraged from smoking in comparison to young adults who do not smoke [65]. Although ***defensive avoidance*** could be viewed as a negative reaction to warning labels, the literature points out that avoidance is a positive outcome that is associated with more thinking about the harms of smoking [54, 66–68]. For example, in a study with a low SES population in the US, researchers found that intention to avoid and ignore graphic warning labels on tobacco packages, was associated with future intentions to search for more information about the harms of smoking [68].

It is possible that the warning label causes adults who smoke to reduce their risk perceptions due to experiencing cognitive dissonance. Glock and Kneer (2009) measured smoking related health risk beliefs before and after exposure to cigarette warning labels. They found that smoking adults’ health risk beliefs were lower following exposure [69]. Another explanation might be that adults who smoke have lower perceived susceptibility than those who do not smoke. For example, in a focus group study, smokers expressed how they are unlikely to experience the negative health of smoking, after being exposed to messages that depict the negative effects of smoking on smokers and others [59]. These findings suggest that fear-evoking messages might have more potential in influencing those who do not smoke or those who are quitting smoking.

Warnings about the impact of e-cigarettes on the cognitive development of youth, addiction, and unknown effects resulted in ***response efficacy*,** among adult focus group participants, meaning that the participants found these warnings to be credible or true, but warnings about toxins and health effects generated ***response inefficacy*** ideas, such as e-cigarettes being just as bad as cigarettes. Responses to health-effect focused warnings among adults were generally expressions of behavioral intentions to quit using these products (**danger control**). Although not explicitly stated in any of the warnings we tested, some adults drew incorrect conclusion that these products are more dangerous than combustible cigarettes (**response inefficacy).** Some adults expressed their belief that warnings of “health effects” would not necessarily impact their use of these products (**fear control via defensive avoidance**). Research shows that people perceive e-cigarettes to be less harmful and less addictive than combustible cigarettes [34, 35, 70]. Therefore, it is somewhat concerning that some of our warnings negatively influenced relative risk perceptions between e-cigarettes and cigarettes.

The set of “unknown risk” warnings tested in this study contained messages suggesting that FDA research has not yet determined the safety of e-cigarettes and that the true extent of potential harms of these products are still unknown. These warnings were seen as particularly troubling and scary to adults (***response efficacy***) and some female youth, leading some participants to question their future use of these products (***danger control***). In response to warnings suggesting unknown effects of these products, some male youth participants even expressed the belief that uncertainty about harms was tantamount to a safe product (***response inefficacy***). Previous research has reported on the differential impact of the suggestion of known versus unknown risk warnings [39]. Specifically, in a study by Kong et al., (2016) messages suggesting uncertainty about ingredients of e-cigarettes was found to increase perceived risk of e-cigarettes, support for e-cigarette control, and lowered self-exempting beliefs and intentions to use e-cigarettes [40]. Previous research has not reported on the differential effects of uncertainty warnings among individuals of different ages or gender. This area of study suggests a potentially important area for future research.

For youth, while promising evidence of response efficacy and danger control emerged among youth exposed to messages in all warning categories but one–addiction—unproductive reactions indicative of fear control were also prevalent among youth respondent across most warning types. On average, youth were more skeptical than adults about the harms of using e-cigarettes. Comments indicative of fear control (***psychological reactance and defensive avoidance)*** were observed from youth in response to warnings in all categories, except the unknown risk category, and were more likely to be voiced by male participants. More research is needed to examine whether male and female youth respond differently to fear-evoking warning messages. Furthermore, while comments indicative of ***response inefficacy*** was found among both youth and adult focus group participants (misconstruing the communicated message), these types of responses were more prevalent among youth groups.

Current research is inconclusive regarding sex and ***gender differences in response*** to tobacco warnings. For instance, a meta-analysis study found that demographic characteristics such as sex and gender do not influence how fear appeal messages are processed [71]. However, a study among Chinese youth and young adults found that fear appeals were more effective with females compared to males, by influencing their protection behavior motivation [72]. It is important to note that the sample in this study was small, which brings to question whether these results accurately represent differences between males and females. Further research is needed to examine whether response to tobacco warning messages have differential effects on male and female youth.

Finally, given that the current FDA-mandated warning label focuses solely on addiction, it is worth noting that we did not see evidence of danger control when participants, adults and youth, discussed warnings about addiction.

### Policy implications

E-cigarettes, which are relatively new products on the market, pose challenges to regulators regarding the communication of health risks. Science on the health impacts of e-cigarettes on the body is still evolving, making definitive warning statements regarding the health risks of using these products challenging. Furthermore, the rapid uptake of e-cigarette use by youth presents challenges in communicating health risks to a population that has not been exposed to decades-long health warnings on combustible cigarette packages. Unlike adults, youth are still evolving in their ability to estimate risks and process the potential future impact of e-cigarette use on health and well-being.

Findings from this study suggest that e-cigarette warnings emphasizing the toxic ingredients and negative health effects of using these products hold the potential to discourage adult who smoke combustible cigarette from transitioning to e-cigarettes. Adults either provided counterarguments to these messages or misinterpreted them. From a public health perspective, this possibility presents a particularly challenging problem. As evidence evolves regarding the harm reduction potential of e-cigarettes, it is possible that warnings communicating “harm reduction” may be more effective at encouraging adult who smoke to switch to e-cigarettes, though this remains an empirical question.

Findings from this study also suggest that e-cigarette warnings emphasizing the toxic ingredients and negative health effects of using e-cigarettes might be less effective in discouraging youth from using these products. Warnings in these categories were more likely to generate fear control responses. It is not clear that such messages are sufficient deterrents for youth, as many dismissed the risk or misinterpreted the message, so a net public health gain is unclear. Considering reactions of youth to warnings across all categories, it appears that warnings regarding the impact of e-cigarette use on their future cognitive development, memory, and mood might offer the strongest potential for deterring product use.

### Limitations

In evaluating the study findings, it is important to note several limitations of the study approach and resultant data collection. First, warning categories contained varying numbers of warnings: toxins (7 warnings); health effects (2 warnings); cognitive development (2 warnings); addiction (4 warnings); and unknown risks (3 warnings). Not all participants viewed all warnings in each category, but all participants viewed a minimum of 1 warning from each category. What we note are patterns in participant responses to the five types of warning statements across all participants.

Youth were often less forthcoming than adults during the focus group interviews. We do not have a way to indicate nonresponse (a component of EPPM) due to the nature of the transcripts we received. During the focus groups, the discussion of the randomized warnings was sometimes rushed at the end of the session, so there was not always equal time provided to each warning viewed.

Finally, we assigned statements to one of three broad categories. There are some in the fear control categorization that could be argued to contain elements of response inefficacy instead. Experimental or survey designs can further extend this exploration of reactions.

### Future research

Warnings are useful to inform a wide audience about the dangers associated with risky behaviors and to raise awareness about the benefits of performing healthy behaviors [73]. Nevertheless, the effectiveness of health warnings may be undermined by resistance among some targeted audiences. Like findings in this study, other studies have shown that health messages can provoke defensive reactions and lead to the recommended behaviors being rejected, especially by those most at risk [74]. Warning messages specifically mentioning youth susceptibility might be met with substantial resistance, (especially among male youth, as we found in our study). All transcripts are available for review in Qualitative Data Repository [75].

Future research efforts should examine the relative effectiveness of these and other warnings to encourage smoking cessation and deter product uptake. Findings from this research indicate that the relative effectiveness will be different for adults and youth, and these impacts could vary by their smoking status. Qualitative findings from the study provide some guidance in terms of likely impact of different warning claims. Future experimental studies of reactions to such warnings can provide further insight into the strength and consistency of these relationships. Furthermore, experimental studies are needed with at-risk populations (adults who smoke or switched to e-cigarettes, youth inclined to try or already using e-cigarettes) to determine the types of warnings likely to engender this type of resistance and which types prompt responses that best promote individual and public health.

The warnings used in this study focused exclusively on the risks and harms of e-cigarette use, yet responses from adult participants in the focus groups, especially those who currently smoke combustible cigarettes, elicited risk comparisons between e-cigarette use and combustible cigarette use. As scientific evidence evolves regarding the potential for reduced harm experienced by adults who smoke combustible cigarettes who switched to e-cigarettes, an important area for future research would be an experimental examination of the effectiveness of “reduced harm” warnings for these products.

## Supporting information

S1 Appendix Study discussion guide. (DOCX)

S2 Appendix Summary of results. (DOCX)

## References

[1] FDA. E-cigarettes, vapes, and other electronic nicotine delivery systems (ENDS) [Internet]. 2022. Available from: https://www.fda.gov/tobacco-products/products-ingredients-components/e-cigarettes-vapes-and-other-electronic-nicotine-delivery-systems-ends

[2] FDA. The real cost campaign [Internet]. 2022. Available from: https://www.fda.gov/tobacco-products/public-health-education-campaigns/real-cost-campaign

[3] FDA. How the FDA is regulating e-cigarettes [Internet]. 2022. Available from: https://www.fda.gov/news-events/fda-voices/how-fda-regulating-e-cigarettes

[4] FDA. Deeming Tobacco Products To Be Subject to the Federal Food, Drug, and Cosmetic Act, as Amended by the Family Smoking Prevention and Tobacco Control Act; Restrictions on the Sale and Distribution of Tobacco Products and Required Warning Statements for Tobacco Products [Internet]. Federal Register. 2016 [cited 2019 Jan 28]. Available from: https://www.federalregister.gov/documents/2016/05/10/2016-10685/deeming-tobacco-products-to-be-subject-to-the-federal-food-drug-and-cosmetic-act-as-amended-by-the 27192730

[5] American Lung Association. The impact of e-cigarettes on the lungs [Internet]. American Lung Association. 2020. Available from: https://www.lung.org/quit-smoking/e-cigarettes-vaping/impact-of-e-cigarettes-on-lung

[6] FDA. Youth tobacco use: Results from the National Youth Tobacco Survey [Internet]. 2020. Available from: https://www.fda.gov/tobacco-products/youth-and-tobacco/youth-tobacco-use-results-national-youth-tobacco-survey

[7] Balfour DJK, Benowitz NL, Colby SM, Hatsukami DK, Lando HA, Leischow SJ, et al. Balancing consideration of the risks and benefits of e-cigarettes. Am J Public Health. 2021 Sep;111(9):1661–72. doi: 10.2105/AJPH.2021.306416 34410826 PMC8589069

[8] Campus B, Fafard P, St. Pierre J, Hoffman SJ. Comparing the regulation and incentivization of e-cigarettes across 97 countries. Soc SciMed. 2021 Dec;291:114187. doi: 10.1016/j.socscimed.2021.114187 34763132

[9] Huang J, Feng B, Weaver SR, Pechacek TF, Slovic P, Eriksen MP. Changing Perceptions of Harm of e-Cigarette vs Cigarette Use Among Adults in 2 US National Surveys From 2012 to 2017. JAMA Netw Open. 2019 Mar 29;2(3):e191047. 30924893 10.1001/jamanetworkopen.2019.1047 PMC6450305

[10] Kozlowski Lynn T, Sweanor D. Withholding differential risk information on legal consumer nicotine/tobacco products: The public health ethics of health information quarantines. Int J Drug Policy. 2016 Jun;32:17–23. doi: 10.1016/j.drugpo.2016.03.014 27209528

[11] General Surgeon. The facts on e-cigarette use among youth and young adults [Internet]. 2021. Available from: https://e-cigarettes.surgeongeneral.gov

[12] National Academies of Sciences, Engineering, and Medicine. Public health consequences of e-cigarettes. Washington, DC: National Academies Press; 2018. 29894118

[13] Cullen KA, Ambrose BK, Gentzke AS, Apelberg BJ, Jamal A, King BA. *Notes from the Field*: Use of Electronic Cigarettes and Any Tobacco Product Among Middle and High School Students—United States, 2011–2018. MMWR Morb Mortal Wkly Rep. 2018 Nov 16;67(45):1276–7. 30439875 10.15585/mmwr.mm6745a5 PMC6290807

[14] Cullen KA, Gentzke AS, Sawdey MD, Chang JT, Anic GM, Wang TW, et al. E-cigarette use among youth in the United States, 2019. JAMA. 2019 Dec 3;322(21):2095. doi: 10.1001/jama.2019.18387 31688912 PMC6865299

[15] Park-Lee E, Ren C, Sawdey MD, Gentzke AS, Cornelius M, Jamal A, et al. *Notes from the field*: E-cigarette use among middle and high school students—National Youth Tobacco Survey, United States, 2021. MMWR Morb Mortal Wkly Rep. 2021 Oct 1;70(39):1387–9. 34591834 10.15585/mmwr.mm7039a4 PMC8486384

[16] Cooper M, Park-Lee E, Ren C, Cornelius M, Jamal A, Cullen KA. *Notes from the Field*: E-cigarette Use Among Middle and High School Students—United States, 2022. MMWR Morb Mortal Wkly Rep. 2022 Oct 7;71(40):1283–5. 36201370 10.15585/mmwr.mm7140a3 PMC9541029

[17] Witte K. Putting the fear back into fear appeals: The extended parallel process model. Commun Monogr. 59:329–49.

[18] Witte K. Fear as motivator, fear as inhibitor: Using the extended parallel process model to explain fear appeal successes and failures. In: Anderson PA, Guerrero LK, editors. Handbook of communication and emotion: Research, theory, applications, and contexts. San Diego: CA: Academic Press; 1998. p. 423–50.

[19] Greiner Safi A, Kalaji M, Avery R, Niederdeppe J, Mathios A, Dorf M, et al. Examining perceptions of uncertain language in potential e-cigarette warning labels: Results from 16 focus groups with adult tobacco users and youth. Health Commun. 2023. 10.1080/10410236.2023.2170092 36717390 PMC10387126

[20] American Lung Association. E-cigarettes [Internet]. 2021. Available from: https://www.lung.org/quit-smoking/e-cigarettes-vaping/lung-health

[21] Centers for Disease Control and Prevention. Substance use during pregnancy [Internet]. 2020. Available from: https://www.cdc.gov/reproductivehealth/maternalinfanthealth/substance-abuse/substance-abuse-during-pregnancy.htm

[22] CDC. Quick facts on the risks of e-cigarettes for kids, teens, and young adults [Internet]. 2021 [cited 2021 Jul 20]. Available from: https://www.cdc.gov/tobacco/basic_information/e-cigarettes/Quick-Facts-on-the-Risks-of-E-cigarettes-for-Kids-Teens-and-Young-Adults.html

[23] U.S. Department of Health and Human Services. E-cigarette use among youth and young adults: A report of the Surgeon General [Internet]. Atlanta, GA: U.S. Department of Health and Human Services, Centers for Disease Control and Prevention, National Center for Chronic Disease Prevention and Health Promotion, Office on Smoking and Health; 2016. Available from: https://www.cdc.gov/tobacco/data_statistics/sgr/e-cigarettes/pdfs/2016_sgr_entire_report_508.pdf

[24] Kim HS, Kim YJ, Seo YR. An Overview of Carcinogenic Heavy Metal: Molecular Toxicity Mechanism and Prevention. J Cancer Prev. 2015 Dec 30;20(4):232–40. doi: 10.15430/JCP.2015.20.4.232 26734585 PMC4699750

[25] Lee HW, Park SH, wen Weng M, Wang HT, Huang WC, Lepor H, et al. E-cigarette smoke damages DNA and reduces repair activity in mouse lung, heart, and bladder as well as in human lung and bladder cells. PNAS. 2018 Feb 13;115(7):E1560–9. doi: 10.1073/pnas.1718185115 29378943 PMC5816191

[26] Centers for Disease Control and Prevention. Transcript of CDC Telebriefing: Update on Lung Injury Associated with E-cigarette Product Use, or Vaping [Internet]. 2019. Available from: https://www.cdc.gov/media/releases/2019/t0919-lung-inury-vaping.html

[27] American Cancer Society. Health risks of e-cigarettes [Internet]. 2020. Available from: https://www.cancer.org/healthy/stay-away-from-tobacco/health-risks-of-tobacco/health-risks-of-e-cigarettes.html

[28] FDA Commissioner O of the. Press Announcements—Statement from FDA Commissioner Scott Gottlieb, M.D., on new steps to address epidemic of youth e-cigarette use [Internet]. 2018 [cited 2019 Jan 28]. Available from: https://www.fda.gov/NewsEvents/Newsroom/PressAnnouncements/ucm620185.htm

[29] Surgeon General. The Health Consequences of Smoking—50 Years of Progress: A Report of the Surgeon General, 2014 | SurgeonGeneral.gov [Internet]. 2014 [cited 2019 Feb 12]. Available from: https://www.surgeongeneral.gov/library/reports/50-years-of-progress/index.html

[30] Health People. Tobacco [Internet]. 2020. Available from: https://www.healthypeople.gov/2020/leading-health-indicators/2020-lhi-topics/Tobacco

[31] FDA. FDA permits marketing of e-cigarette products, marking first authorization of its kind by the agency [Internet]. 2021. Available from: https://content.govdelivery.com/accounts/USFDA/bulletins/2f72348

[32] Katz SJ, Lindgren B, Hatsukami D. E-cigarettes Warning Labels and Modified Risk Statements: Tests of Messages to Reduce Recreational Use. Tob Regul Sci. 2017 Oct 1;3(4):445–58. doi: 10.18001/TRS.3.4.6 30238022 PMC6141046

[33] Mays D, Smith C, Johnson AC, Tercyak KP, Niaura RS. An experimental study of the effects of electronic cigarette warnings on young adult nonsmokers’ perceptions and behavioral intentions. Tob Induc Dis. 2016 May 26;14(1):17. 27231479 10.1186/s12971-016-0083-x PMC4880975

[34] Berry C, Burton S, Howlett E. The impact of e-cigarette addiction warnings and health-related claims on consumers’ risk beliefs and use intentions. J Public Policy Mark. 2017;36(1):54–69.

[35] Berry C, Burton S, Howlett E. Are cigarette smokers’, e-cigarette users’, and dual users’ health-risk beliefs and responses to advertising influenced by addiction warnings and product type? Nicotine Tob Res. 2017 Oct 1;19(10):1185–91. doi: 10.1093/ntr/ntx075 28379568

[36] Andrews JC, Mays D, Netemeyer RG, Burton S, Kees J. Effects of e-cigarette health warnings and modified risk ad claims on adolescent e-cigarette craving and susceptibility. Nicotine Tob Res. 2019 May 21;21(6):792–8. doi: 10.1093/ntr/nty076 29669064

[37] Wackowski O, Sontag J, Hammond D, O’Connor R, Ohman-Strickland P, Strasser A, et al. The impact of e-cigarette warnings, warning themes and inclusion of relative harm statements on young adults’ e-cigarette perceptions and use intentions. IJERPH. 2019 Jan 10;16(2):184. doi: 10.3390/ijerph16020184 30634618 PMC6352031

[38] Noar SM, Rohde JA, Horvitz C, Lazard AJ, Cornacchione Ross J, Sutfin EL. Adolescents’ receptivity to E-cigarette harms messages delivered using text messaging. Addict Behav. 2019 Apr 1;91:201–7. doi: 10.1016/j.addbeh.2018.05.025 29960716 PMC6275144

[39] Owusu D, Massey Z, Popova L. An experimental study of messages communicating potential harms of electronic cigarettes. Cummings M, editor. PLoS ONE. 2020 Oct 21;15(10):e0240611. doi: 10.1371/journal.pone.0240611 33085686 PMC7577451

[40] Kong G, Cavallo DA, Camenga DR, Morean ME, Krishnan-Sarin S. Preference for gain- or loss-framed electronic cigarette prevention messages. Addict Behav. 2016 Nov;62:108–13. doi: 10.1016/j.addbeh.2016.06.015 27344117 PMC5512445

[41] Zhao X, Fink EL. Proattitudinal versus counterattitudinal messages: Message discrepancy, reactance, and the boomerang effect. Commun Monog. 2021 Jul 3;88(3):286–305.

[42] Richards AS, Bessarabova E, Banas JA, Bernard DR. Reducing psychological reactance to health promotion messages: comparing preemptive and postscript mitigation strategies. Health Commun. 2022 Feb 23;37(3):366–74. doi: 10.1080/10410236.2020.1839203 33106046

[43] Goodman EP. Visual gut punch: persuasion, emotion, and the constitutional meaning of graphic disclosure. Cornell Law Review. 2014;99(3):513–70. 24745102

[44] Sawicki NN. Compelling image: The constitutionality of emotionally persuasive health campaigns. Maryland Law Review. 2014;73(2):458–522.

[45] C+R Research [Internet]. Available from: https://www.crresearch.com/methods

[46] Pérez A, Bluestein MA, Kuk AE, Chen B. Age of e-cigarette initiation in USA young adults: Findings from the Population Assessment of Tobacco and Health (PATH) study (2013–2017). Washio Y, editor. PLoS ONE. 2021 Dec 13;16(12):e0261243. doi: 10.1371/journal.pone.0261243 34898629 PMC8668126

[47] Gentzke AS, Creamer M, Cullen KA, Ambrose BK, Willis G, Jamal A, et al. Tobacco Product Use Among Middle and High School Students—United States, 2011–2018 [Internet]. U.S. Department of Health and Human Services, Centers for Disease Control and Prevention; 2019 Feb [cited 2021 Oct 26] p. 157–64. (MMWR). Report No.: 68(6). Available from: http://www.cdc.gov/mmwr/volumes/68/wr/mm6806e1.htm?s_cid=mm6806e1_w

[48] Villarroel MA, Cha AE, Vahratian A. Electronic cigarette use among U.S. Adults, 2018 [Internet]. Center for Disease Contol and Prevention; 2020. (NCHS Data Brief). Report No.: 365. Available from: https://www.cdc.gov/nchs/data/databriefs/db365-h.pdf 32487293

[49] Stallings-Smith S, Ballantyne T. Ever Use of E-Cigarettes Among Adults in the United States: A Cross-Sectional Study of Sociodemographic Factors. Inquiry. 2019 Jan;56:004695801986447. doi: 10.1177/0046958019864479 31328601 PMC6647205

[50] Patton M. Qualitative research and evaluation methods. 4th ed. Thousand Oaks, CA: Sage Publications; 2015.

[51] Percy W, Kostere K, Kostere S. Generic Qualitative Research in Psychology. TQR [Internet]. 2015 Feb 16 [cited 2021 Oct 26]; Available from: https://nsuworks.nova.edu/tqr/vol20/iss2/7/

[52] Ramanadhan S, Revette AC, Lee RM, Aveling EL. Pragmatic approaches to analyzing qualitative data for implementation science: an introduction. Implement Sci Commun. 2021 Dec;2(1):70. doi: 10.1186/s43058-021-00174-1 34187595 PMC8243847

[53] Chapman A, Hadfield M, Chapman C. Qualitative research in healthcare: An introduction to grounded theory using thematic analysis. JR Coll Physicians Edinb. 2015 Sep 1;45(3):201–5. doi: 10.4997/JRCPE.2015.305 26517098

[54] Kees J, Burton S, Andrews JC, Kozup J. Understanding How Graphic Pictorial Warnings Work on Cigarette Packaging. J Public PolicyMark. 2010 Nov 1;29(2):265–76.

[55] Byrne S, Katz SJ, Mathios A, Niederdeppe J. Do the ends justify the means? A test of alternatives to the FDA proposed cigarette warning labels. Health Commun. 2015 Jul 3;30(7):680–93. doi: 10.1080/10410236.2014.895282 25119144

[56] Noar SM, Hall MG, Francis DB, Ribisl KM, Pepper JK, Brewer NT. Pictorial cigarette pack warnings: a meta-analysis of experimental studies. Tob Control; London. 2016 May;25(3):341. doi: 10.1136/tobaccocontrol-2014-051978 25948713 PMC4636492

[57] Noar SM, Francis DB, Bridges C, Sontag JM, Ribisl KM, Brewer NT. The impact of strengthening cigarette pack warnings: Systematic review of longitudinal observational studies. Soc SciMed. 2016 Sep 1;164:118–29. doi: 10.1016/j.socscimed.2016.06.011 27423739 PMC5026824

[58] Schneider S, Gadinger M, Fischer A. Does the effect go up in smoke? A randomized controlled trial of pictorial warnings on cigarette packaging. Patient EduCouns. 2012 Jan;86(1):77–83. 10.1016/j.pec.2011.03.005 21482060

[59] Mead EL, Cohen JE, Kennedy CE, Gallo J, Latkin CA. The role of theory-driven graphic warning labels in motivation to quit: a qualitative study on perceptions from low-income, urban smokers. BMC Public Health. 2015 Dec;15(1):92. doi: 10.1186/s12889-015-1438-6 25880277 PMC4349464

[60] Mead EL, Cohen JE, Kennedy CE, Gallo J, Latkin CA. The influence of graphic warning labels on efficacy beliefs and risk perceptions: a qualitative study with low-income, urban smokers. Tob Induced Dis. 2016 Dec;14(1):25. doi: 10.1186/s12971-016-0088-5 27471440 PMC4964038

[61] Owusu D, So J, Popova L. Reactions to tobacco warning labels: predictors and outcomes of adaptive and maladaptive responses. AddictResTheory. 2019 Sep 3;27(5):383–93. doi: 10.1080/16066359.2018.1531127 31511769 PMC6738943

[62] Weinstein ND. Accuracy of smokers’ risk perceptions. Ann Bev Med. 1998;6. doi: 10.1007/BF02884459 9989319

[63] Weinstein ND, Marcus SE, Moser RP. Smokers’ unrealistic optimism about their risk. Tob Control. 2005 Feb 1;14(1):55–9. doi: 10.1136/tc.2004.008375 15735301 PMC1747991

[64] Dillard JP, Shen L, Vail RG. Does Perceived Message Effectiveness Cause Persuasion or vice Versa? 17 Consistent Answers. Hum Commun Res. 2007 Oct 1;33(4):467–88.

[65] Cameron LD, Pepper JK, Brewer NT. Responses of young adults to graphic warning labels for cigarette packages. Tob Control. 2013;24(e1):e14–22. doi: 10.1136/tobaccocontrol-2012-050645 23624558 PMC3884029

[66] Yong HH, Borland R, Thrasher JF, Thompson ME, Nagelhout GE, Fong GT, et al. Mediational pathways of the impact of cigarette warning labels on quit attempts. Health Psychol. 2014 Nov;33(11):1410–20. doi: 10.1037/hea0000056 24977309 PMC4600667

[67] Peters E, Romer D, Slovic P, Jamieson KH, Wharfield L, Mertz CK, et al. The impact and acceptability of Canadian-style cigarette warning labels among U.S. smokers and nonsmokers. Nicotine Tob Res. 2007 Apr;9(4):473–81. doi: 10.1080/14622200701239639 17454702

[68] McCloud RF, Okechukwu C, Sorensen G, Viswanath K. Cigarette graphic health warning labels and information avoidance among individuals from low socioeconomic position in the U.S. Cancer Causes Control. 2017 Apr;28(4):351–60. doi: 10.1007/s10552-017-0875-1 28255678

[69] Glock S, Kneer J. Are Deterrent Pictures Effective? The Impact of Warning Labels on Cognitive Dissonance in Smokers. Appl Psychol Health Well-Being. 2009 Sep;1(3):356–73.

[70] Czoli CD, Fong GT, Mays D, Hammond D. How do consumers perceive differences in risk across nicotine products? A review of relative risk perceptions across smokeless tobacco, e-cigarettes, nicotine replacement therapy and combustible cigarettes. Tob Control. 2017 Mar;26(e1):e49–58. doi: 10.1136/tobaccocontrol-2016-053060 27625408

[71] Witte K, Allen M. A meta-analysis of fear appeals: Implications for effective health campaigns. Health Educ Behav. 2000;27:591–615. 11009129 10.1177/109019810002700506

[72] Sun C, Wang F, Jiang M. How Can E-Cigarette Fear Appeals Improve the Perceived Threat, Fear, Anger, and Protection Motivation of Young People. Front Psychol. 2021 Aug 30;12:676363. doi: 10.3389/fpsyg.2021.676363 34526929 PMC8435607

[73] Blondé J, Easterbrook MJ, Harris PR, Girandola F, Khalafian A. Taking advantage of multiple identities to reduce defensiveness to personally threatening health messages. Appl Psychol Health Well Being. 2022 Mar 8;aphw.12355. doi: 10.1111/aphw.12355 35259289

[74] van ‘t Riet J, Ruiter RAC. Defensive reactions to health-promoting information: an overview and implications for future research. Health Psychol Rev. 2013 May 1;7(sup1):S104–36.

[75] Avery R J, Byrne S, Dorf MC, Greiner Safi A, Kalaji M, Mathios AD, et al. Youth and adult responses to e-cigarette warning messages—focus group transcripts [data set]. Qualitative Data Repository. 2023. 10.5064/F6LQGCLG

