# Supplementary material for: Perceived threat and fear responses to e-cigarette warning label messages: Results from 16 focus groups with U.S. youth and adults
Source: PLoS One. 2023 Jun 23;18(6):e0286806. doi: 10.1371/journal.pone.0286806 (PMC10289367; doi:10.1371/journal.pone.0286806)
Supplement: S1 Appendix — (DOCX) [file pone.0286806.s001.docx]

S1 Appendix. Study Discussion Guide

**Section 1: Memorable advertising**

To get us started, I’d like to understand **where you see advertising**.

- What are the places you can think of where you see or hear ads?
  - Can be at home, on the go, etc…what comes to mind? *(Listen/probe for TV, online, video streaming, billboards, print, radio, etc.)*
  - What do you recall having seen recently? What was the message?
  - Can you think of any ads that have really “stuck” with you?
    - What was that, and why was it so effective?
    - Did you ultimately do what it suggested?

**Section 2: Understand e-cigarette perceptions**

Now let’s talk about e-cigarettes and tobacco vaping products like JUUL, N-Joy, Blu, Vuse, Puffs, Posh and other products like this. *(If necessary, clarify this excludes THC products and hookah.)*

- *(Write on notepad the first three (3) words that come to mind when you think about vaping.)*
- What are e-cigarettes?
- What have you heard about them?
- What words did you write down?
  - Why do you think those things came to mind?

**Section 3: Advertising strategy evaluation**

The next thing we’ll do is look at some more fully developed advertisements. For each one, I’ll read any text aloud while you read it silently. Then, you’ll circle any images, words, or phrases that are appealing in some way, and cross out anything that you dislike. Finally, you’ll give each one a score of 1-10 for how convincing it is, 1 being worst and 10 being best.

- For each strategy:
  - What is this **trying to tell you?**
    - **What elements communicate that to you?**
  - What **score of 1-10** did you write down? **What contributed to that?**
  - What did you circle—**what did you like**? Why?
  - What did you cross out—**what did you dislike**? Why?

**Section 4: Individual risk claim evaluation**

Now, I’m going to show you several different statements that health officials are thinking about requiring e-cigarette advertisements to include. [SHOW THEM THE WARNING FROM ONE OF THE PREVIOUS E-CIGARETTE ADS to walk them through where this would go, and that these would be in addition to the one that is currently there]. They are really interested in your opinion about these statements. Each one has a two-letter label that doesn’t mean anything, it just helps me tell them apart. *[Expose in 4 rounds of 2 statements apiece.]*

For each one, I’ll read it aloud while you read it silently. Then, you’ll circle any words or phrases that are appealing in some way and cross out any words or phrases that you dislike.

- For each statement:
  - What is this **trying to tell you?**
  - What did you circle—**what did you like**? Why?
  - What did you cross out—**what did you dislike**? Why?
  - What does this **suggest you may want to do, or not do**? **How convincing is it?** Please give it a score of 1-10 where 1 is least convincing and 10 is most convincing.

**Section 5: Comparison of risk claims**

Now that you’ve seen all of these, you may rethink the scores you’ve given to each one. Now let’s work together as a group to arrange them in a row on the center of the table from “least convincing” to “most convincing.” I’ve got index cards for each end of the scale. Please discuss together where each belongs, and your reasoning why. Place each where they belong; you may have “ties” if some seem truly equal to you as a group. After you’re mostly satisfied with the arrangement, I’ll ask you to explain to me why each is where it is, and I’ll invite anyone who respectfully disagrees to speak up.

- Okay, is everyone reasonably satisfied? I’ll read out the order of the labels just for the tape recording.
- Now, please tell me **why these closest to “most convincing” belong there. What’s in the middle and why? What’s “least convincing” and why?**
- Were there **any ties**? What made those happen?
- What were any **points of debate** *(bringing up anything overheard)* while you were moving them around? What was that about, and how did you resolve it?
- How are you thinking about e-cigarettes now?
- How are you thinking about other kinds of tobacco products like cigarettes now?

Section 6: Wrapping up

COMPLETE THE STATEMENT:

If I am using an e-cigarette, it’s because….

If am NOT using an e-cigarette, it’s because
