# Supplementary material for: Perceived threat and fear responses to e-cigarette warning label messages: Results from 16 focus groups with U.S. youth and adults
Source: PLoS One. 2023 Jun 23;18(6):e0286806. doi: 10.1371/journal.pone.0286806 (PMC10289367; doi:10.1371/journal.pone.0286806)
Supplement: S2 Appendix — (DOCX) [file pone.0286806.s002.docx]

S2. Appendix. Summary of results.

| **Warning Category** | **Emotional Response Type** |
| --- | --- |
| **TOXINS** |  |
| **Toxins – Adults** |  |
| - The chemical names and health consequences communicated in these warnings was seen as alarming, and a deterrent to using these products | Danger control |
| - After seeing these warnings, participants drew the conclusion that e-cigarettes are no better for you than combustible cigarettes | Response inefficacy |
| - Among a few adult combustible cigarette smokers, these warnings generated counter arguments regarding the veracity of whether lung damage was irreversible | Fear control (*psychological reactance*) |
| **Toxins – Youth** |  |
| - Warnings using the words ‘toxic’ and the use of ‘chemical names’ elicited intentions to avoid product use among youth | Danger control |
| - Warnings using the word ‘irreversible’ generated response efficacy statements among some male youth | Response efficacy |
| - Some male youth viewing these warnings developed counter arguments to the message | Fear control (*psychological reactance)* |
| - Some male youth viewing these warnings believe they would not be read by young people | Fear control (d*efensive avoidance*) |
| **HEALTH EFFCTS** |  |
| **Health Effects – Adults** |  |
| - Warnings with the word ‘cancer’ or ‘irreversible lung damage’ led to beliefs that these products are no better than combustible cigarettes | Response inefficacy |
| - Warnings with the word ‘cancer’ or ‘irreversible lung damage’ elicited statements of behavioral intentions to quit | Danger control |
| - Participants who had already switched to e-cigarettes did not believe these warnings would have an impact on their use/quitting behavior | Fear control (d*efensive avoidance*) |
| **Health Effects – Youth** |  |
| - Youth exposed to these warnings found them powerful and scary, suggesting they may deter initiation of product use | Danger control |
| - Some male youth focus group participants developed counter arguments to these warnings | Fear control (*psychological reactance)* |
| - In response to the warning saying simply, “health risk to young people,” some youth noted that this is a message they had heard many times before and it was no longer impactful on their decision to use or not use the product | Fear control (d*efensive avoidance*) |
| - Some male youth exposed to the warning referencing ‘health risks to young people’ inferred that these products do not offer risks to other demographic groups | Response inefficacy |
| **COGNITIVE DEVELOPMENT** |  |
| **Cognitive development – Adults** |  |
| - Adult participants found these statements to be highly believable and expressed their belief that these warnings would be a strong deterrent for the use of these products by young people | Response efficacy |
| **Cognitive development – Youth** |  |
| - The claim in the e-cigarette warning of a connection between smoking e-cigarettes and brain development and long-term memory and mood impacts was accepted as true by almost all youth focus group participants | Response efficacy |
| - Some youth believed that this warning might deter young people from initiating e-cigarette use | Danger control |
| - Some female youth reported that they had heard of the warning of a connection between e-cigarette use and memory issues so frequently that the warning no longer held any meaning for them | Fear control (*defensive avoidance*) |
| **ADDICTION** |  |
| **Addiction – Adults** |  |
| - The warning referencing ‘future addiction’ was perceived as a true statement by adult smokers | Response efficacy |
| - One participant found the comparison to cocaine as inappropriate and overstating the risk | Fear control (*psychological reactance)* |
| **Addiction – Youth** |  |
| - Some youth viewing these warnings concluded that e-cigarettes could indeed lead to other addictions | Response efficacy |
| - Some youth participants argued the addiction message would not be heeded as it was ‘old news to them and such messages would not deter them from using these products | Fear control (d*efensive avoidance*) |
| - A few female youth participants did not believe that e-cigarettes would be a gateway drug to harder illegal drugs such as cocaine | Fear control (d*efensive avoidance*) |
| **UNKNOWN RISKS** |  |
| **Unknown risks – Adults** |  |
| - Several adults found these warnings troubling and wondered about future health impacts | Response efficacy |
| **Unknown risks – Youth** |  |
| - Some female participants expressed that this warning might be a deterrent to using these products due to the unknown effects on the body | Danger control |
| - Some male youth participants expressed that if effects have not been identified so far, the products were probably safe | Response inefficacy |
